# A Narrative Review of Long-Term Outcomes and Imaging-Based Failure Analysis in Lateral Unicompartmental Knee Arthroplasty

**DOI:** 10.7759/cureus.102488

**Published:** 2026-01-28

**Authors:** Hassan Alherz, Ahmad N Boeisa, Ibrahim M Algahtani, Maria S Abosbaih, Basmah A Alanazi, Abdulaziz A AlSuwilem, Layana N Alothman, Basel Y Almas, Ali N Alabdullah, Faisal D Alshehri

**Affiliations:** 1 Medicine, Sechenov University, Moscow, RUS; 2 Pediatric Orthopedic Surgery, Almoosa Specialist Hospital, Al-Ahsa, SAU; 3 Faculty of Medicine, King Abdulaziz University, Jeddah, SAU; 4 Medicine, King Faisal University, Hofuf, SAU; 5 Medicine, Hail University, Hail, SAU; 6 Medicine, King Khalid University, Abha, SAU

**Keywords:** imaging analysis, implant failure, knee arthroplasty, lateral unicompartmental knee arthroplasty, long-term outcomes

## Abstract

Lateral unicompartmental knee arthroplasty (UKA) remains an underutilized treatment option for isolated lateral compartment osteoarthritis, despite growing evidence supporting its effectiveness. Historically, concerns regarding technical complexity, implant design limitations, and higher revision rates limited its adoption. However, advances in implant technology and surgical techniques have substantially improved outcomes. We conducted a comprehensive literature search for studies including outcomes of UKA. Inclusion prioritized but was not limited to studies published in 2020 and onwards, systematic reviews, meta-analyses, and clinical trials. We summarized the available literature on long-term clinical outcomes, survivorship, and failure patterns following lateral UKA, with particular emphasis on the role of imaging-based failure analysis. Contemporary cohort studies and registry data demonstrate excellent survivorship at long-term follow-up comparable to medial UKA and, in appropriately selected patients, total knee arthroplasty. Imaging modalities, including radiography, computed tomography, fluoroscopy-based kinematic analysis, and magnetic resonance imaging, play a critical role in evaluating component positioning and alignment, along with early signs of failure that may not be clinically apparent. These imaging-based assessments have contributed to refinements in surgical planning and patient selection. Overall, modern lateral UKA has evolved into a reliable and durable procedure when performed with meticulous techniques and appropriate imaging-guided evaluation, supporting its expanded role in contemporary knee arthroplasty practice.

## Introduction and background

Unicompartmental knee arthroplasty (UKA) is a procedure that restores function in osteoarthritis (OA) limited to the medial or lateral compartments of the knee. It is ligament- and bone-preserving, with significant literature showing its greater patient satisfaction and improved outcomes. Lateral UKA is performed less than medial UKA, accounting for only about 5-10% of all UKA procedures [1,2]. This is largely because degenerative pathology, such as OA and osteonecrosis, more commonly affects the medial compartment of the knee [3]. In contrast, lateral UKA remains less commonly performed due to both the lower prevalence of isolated lateral compartment disease and the greater technical complexity of the procedure. Anatomical and kinematic differences of the lateral compartment, along with historical implant design limitations, have contributed to higher revision rates for lateral UKA [4,5]. In this context, imaging‑based failure analysis has become a key tool for evaluating lateral UKA implant performance and informing the need for revision. Radiographic studies have shown that alignment outliers correlate with higher revision rates, showing the value of imaging assessment in finding potential failure patterns that may not be evident clinically. Such evaluations help refine surgical techniques and optimize patient selection in lateral UKA [6-8]. The aim of this narrative review is to summarize the long-term outcomes of lateral UKA and highlight the role of imaging-based failure analysis in identifying potential complications and improving surgical decision-making.

## Review

Epidemiology and utilization trends

UKA constitutes a minority of knee arthroplasties in the U.S., accounting for 8-10% of all procedures. However, its use has been rising. For example, one analysis from 2016 reported 7,194 UKAs, versus 128,849 total knee arthroplasties (TKAs), between 2007 and 2016, with annual rates of both procedures increasing over time [9]. More recent data confirm steep growth: a large U.S. registry observed 1,662 UKAs in 2022 (vs. 241 in 2012), a 590% increase [10]. Even with this rise, lateral UKAs remain a small fraction of those cases; one study found a medial-to-lateral UKA ratio of about 14.2:1 [11]. In other words, lateral UKAs comprise roughly 5-7% of UKAs, reflecting both the lower incidence of isolated lateral OA and the more stringent indication criteria [11].

Several economic analyses suggest that UKA can be cost-effective relative to TKA when its durability approaches that of TKA. In a decision-analysis model, Soohoo et al. (2006) found that if lateral UKA survival is only a few years shorter than that of TKA, then UKA yields greater quality-adjusted life years at an incremental cost <$50,000 per QALY. Under these assumptions, UKA is shown to be a cost-effective alternative to TKA [12]. A summary of that work similarly concluded that, with appropriate patient selection and assuming comparable function, UKA is economically favorable to TKA. In practice, cost-effectiveness may vary by health system, but these studies indicate a consistent trend: partial knee replacement can offer good value provided implant longevity is maintained [12].

The knee joint has medial and lateral compartments with distinct anatomy and biomechanics. The lateral compartment is involved in only about 10% of knee OA cases. and has unique kinematics (including a pronounced “screw-home” mechanism during extension), which makes lateral UKA technically demanding [13]. Compared to TKA, UKA offers several well-documented benefits: it preserves the cruciate ligaments and more native bone stock, yielding a more natural-feeling knee with superior range of motion and proprioception [14,15]. Patients undergoing UKA tend to recover faster, experience less early postoperative pain, and report higher quality-of-life scores in the short term [14]. Together, these epidemiologic, economic, and biomechanical considerations underscore why lateral UKA, despite its limited utilization, requires focused evaluation of outcomes and imaging-based failure mechanisms, which is the central aim of this review.

Surgical approaches for TKA and UKA

Unlike medial UKA, lateral UKA can be performed via either a traditional medial parapatellar arthrotomy or a lateral parapatellar approach. A medial approach is familiar to most surgeons and allows easy conversion to TKA, whereas a lateral parapatellar approach provides direct access to the lateral compartment and preserves medial soft tissues. In either case, the tibial cut technique is adjusted for the lateral anatomy: surgeons often use a vertical trans-patellar tibial cut with slight internal rotation of the tibial component to accommodate the lateral knee’s kinematics [16]. Meticulous alignment is critical; improper cuts or component positioning can lead to malalignment or ligament imbalance and early failure. Both fixed- and mobile-bearing prostheses have been used in lateral UKA. Current practice favors fixed-bearing designs for the lateral compartment due to their simpler geometry and lower dislocation risk (see below). Overall, lateral UKA uses less bone resection and preserves soft tissues compared to TKA, contributing to shorter operative time and faster rehabilitation [14,15].

UKA designs and implant evolution

Early lateral UKAs often used mobile-bearing implants (e.g., Oxford domed lateral) but encountered high failure rates due to bearing dislocations. Modern designs have shifted toward fixed-bearing lateral UKA, such as the Oxford Fixed Lateral (FLO). A recent comparative study found fixed-bearing lateral UKA had dramatically better survivorship (98% at ~3-5 years) than matched mobile-bearing cases (79%), with dislocation being the main failure in mobile implants. Importantly, functional outcomes were equivalent between designs, leading authors to recommend fixed-bearing implants for isolated lateral OA [17]. Registry data confirm these findings: Bunyoz et al. (2025) reported that adopting the FLO fixed-bearing implant has significantly reduced lateral UKA revision risk (5-year revision 10.1% overall, declining to 7.3% in recent series) [18]. In short, implant evolution, from early domed mobile bearings to contemporary fixed-bearing prostheses, has markedly improved lateral UKA survival [4].

Indications, contraindications, and patient selection for lateral UKA

Ideal candidates for UKA have isolated, end-stage lateral-compartment OA or spontaneous osteonecrosis in the lateral compartment, with a functionally intact anterior cruciate ligament and essentially normal medial cartilage. Any existing valgus deformity should be fully correctable. Historically, lateral UKA indications were conservative, but modern evidence has broadened them. Notably, factors once thought to contraindicate UKA, such as patellofemoral joint degeneration, patient age, activity level, or obesity, are no longer absolute exclusions. For example, Kennedy et al. found that neither patellofemoral wear nor body weight significantly affected outcomes in lateral UKA, with other studies reporting satisfactory results even in obese or younger patients. In summary, appropriate patient selection (isolated lateral OA, good ligament stability) remains crucial, but with these criteria, patients who are older, heavier, or more active can still do well with modern lateral UKA [16,19].

Lateral versus medial UKA: outcomes and survivorship

Large-scale analyses show that lateral and medial UKA yield comparable clinical results when each is used for its appropriate indication. A meta-analysis of 33,999 medial vs 2,853 lateral UKAs found no significant differences between the two groups in postoperative pain scores, functional outcome scores, or short-/mid-term survival. Both compartments have reported survival rates around 94-96% at 5-10 years. For instance, Han et al. reported 95.6% 5-year survival for medial UKA versus 94.6% for lateral UKA, and 92.8% vs 86.6% at 10+ years (differences were not statistically significant) [3]. Registry data from Italy (RIPO) even showed slightly better 10-year survivorship for lateral UKA (95.2%) than medial UKA (87.5%) [20].

Failure modes do differ somewhat by compartment. In lateral UKA, early failures were mostly due to bearing dislocation when mobile inserts were used. This complication has become rare with fixed-bearing designs [17]. Progression of OA in other compartments (especially the medial compartment or patellofemoral joint) is the leading cause of late lateral UKA failure [20,21]. By contrast, medial UKA historically has been challenged by overcorrection into valgus alignment, which can overload the lateral compartment. Overall, modern series report similarly high survival for both medial and lateral UKAs, showing that with correct technique, lateral UKA is as durable as its medial counterpart [3].

UKA versus TKA: clinical outcomes and adverse events

Compared to TKA, UKA (lateral or medial) generally offers faster recovery, more natural kinematics, and fewer perioperative complications [14,22]. UKA patients typically regain motion and function sooner, have less initial pain and swelling, and report higher early satisfaction [14]. Of note, one study found that the risk of any postoperative complication with UKA is dramatically lower than with TKA, an 82% relative risk reduction (OR≈0.2) for any complication at one year. UKA also halves the risk of serious issues like infection, thromboembolism, or cardiac events compared to TKA [22].

However, UKA tends to have higher revision rates in the long term. National registries consistently show roughly twice the revision rate for UKA versus TKA (often cited as 2-3 times higher), even though mechanical complications like deep infection occur less frequently after UKA [18,22]. In one analysis, five-year revision risk was ~10.1% for lateral UKA versus ~5.0% for TKA. Many factors contribute to this difference (e.g., lower revision threshold for UKA, surgeon experience). Nonetheless, functional outcome scores and patient satisfaction at mid-term follow-up are often similar between well-matched UKA and TKA cohorts. In elderly or lower-demand patients, UKA and TKA have shown comparable survivorship at 5-10 years [14,22]. In summary, UKA offers superior early recovery and fewer early complications, with the trade-off of a somewhat higher chance of needing a revision arthroplasty later on [18,22].

Surgical technologies and implant design impact

Recent technological advances have further improved lateral UKA outcomes. Robotic-assisted lateral UKA, which uses preoperative imaging and robotic guidance for precise bone cuts, has yielded excellent long-term results. For example, Ruderman et al. (2024) reported 96.1% implant survival at 10 years and 94.4% patient satisfaction after robotic-assisted lateral UKA, with very low reported pain [21]. In this series, the few failures were mainly due to OA progression in untreated compartments or unexplained pain, not mechanical implant issues. However, at five-year follow-up, robotic and conventional lateral UKAs have shown similarly excellent outcomes; one study found no significant difference in clinical scores or revision rates between the two methods [23]. Thus, while robotics may improve surgical precision, its long-term advantage over expert conventional techniques is still being defined.

On the implant side, fixed-bearing designs are now strongly preferred in lateral UKA due to their lower failure rates. As discussed above, studies repeatedly show fixed-bearing lateral UKAs achieve higher survivorship than mobile-bearing versions [17]. Newer fixed implants also allow minimally invasive approaches and simplified instrumentation. In sum, modern fixed-bearing prostheses and the adoption of computer assistance have made lateral UKA more reproducible and successful than early iterations.

Failure causes, imaging-based analysis, and registry data

The most common failure after lateral UKA is progressive OA in another compartment of the knee, particularly the medial compartment or patellofemoral joint [20,21]. A variety in reported failure rates due to OA progression is seen, ranging from 1% to 12%, with some studies reporting much higher, up to 26% [24]. A systematic review by Bonanzinga et al. discovered OA progression to account for 34% [25]. Another systematic review found OA as an indication for revision rates to be 41% across cohort studies and 30% among registry studies [26]. Also, a systematic review by Ernstbrunner et al. noted the most common failure mechanisms to be osteoarthritis progression and aseptic loosening, being 30% and 22%, respectively [27]. Other causes include aseptic loosening (usually tibial), bearing dislocation (particularly with mobile designs), and patellar joint instability [19,26-29].

In practice, surgeons review post-op X-rays (AP, lateral, and stress views) to ensure adequate alignment and ligament balance. Imaging plays an essential role in failure analysis: radiographs and advanced imaging are used to assess implant positioning and alignment [30]. Radiographic measurements obtained from standing anteroposterior and lateral knee X-rays, including the mechanical femorotibial angle, mechanical femoral and tibial angles, and posterior tibial slope, can be interpreted using UKA-specific thresholds. In lateral UKA, postoperative alignment goals differ from those of medial UKA and TKA. Although undercorrection of the coronal deformity is generally recommended to avoid overloading the opposite compartment, studies have shown that leaving excessive residual valgus (≤ 3°) after lateral UKA is associated with worse clinical outcomes and lower survivorship, whereas greater correction of the valgus deformity (typically > 4°), without overcorrection into varus, is associated with improved results. The tibial component alignment and posterior tibial slope are generally preserved close to the patient’s native anatomy [31,32]. Malalignment or improper component rotation can accelerate wear or cause ligament imbalance. For example, Alesi et al. showed that accounting for the “screw-home” mechanism by internally rotating the tibial tray (10-15°) helped achieve excellent long-term outcomes [20]. Deviation from these expected UKA-specific alignment targets, or progressive changes compared with early postoperative radiographs, may indicate implant malposition, subsidence, or loosening and serve as radiographic markers of implant failure [24,33,34].

Another radiographic assessment to note is degeneration of the medial compartment, where preservation of this compartment is important to assess before lateral UKA. On the contrary, recent studies have shown that asymptomatic and mild-to-moderate involvement of the patellofemoral joint (PFJ) is not related to poor outcomes [35]. A meta-analysis by Yang et al. showed medial PFJ degeneration and patella cartilage damage do not influence outcomes, although another study suggests otherwise for severe lateral PFJ degeneration [36,37].

Single-plane fluoroscopy with 2D/3D reconstruction can assess lateral UKA kinematics, but accuracy may be lower than for TKA. Van Duren et al. reported high in-plane translational accuracy (<0.5 mm) but larger out-of-plane (4.1 mm) and rotational errors (0.6-2.3°), especially for femoral rotation due to implant symmetry. Despite these limitations, the method is adequate for studying overall knee kinematics and can support image-based failure analysis in lateral UKA, though subtle maltracking may be underestimated [38]. CT‑based imaging allows precise quantification of rotational alignment and implant position that directly affect kinematic behavior. In a preoperative CT analysis, Makhdom et al. demonstrated that misidentification of anatomical landmarks can lead to substantial rotational errors of the tibial component (7.1°), which may contribute to kinematic mismatch and suboptimal contact patterns post‑UKA. These findings support the utility of CT in evaluating rotational parameters that fluoroscopy may underestimate, making it a valuable adjunct for comprehensive imaging‑based failure analysis in lateral UKA [7].

MRI, particularly with metal artifact-reduction techniques, can also be utilized to assess fixation or graft-related failure by directly evaluating the bone-component interface when radiographs are inconclusive. Image-based signs of failure included bone marrow edema pattern adjacent to the implant, fibrous membrane formation along the bone-cement interface, osteolysis, and definitive component loosening characterized by circumferential osseous resorption and/or extensive osteolysis, with or without component displacement [39,40].

Registry and cohort data underscore the durability of modern lateral UKA. Long-term survival rates exceed 90% in most series. For instance, Marullo et al. found 94.7% survival at 15 years in 96 lateral UKAs (with five revisions mainly for other-compartment OA) [41]. Similarly, the Italian registry (Alesi et al.) reported 10-year lateral UKA survival of 95.2% [20]. A large Danish registry analysis (Bunyoz et al.) showed that 10-year revision risk for lateral UKA had fallen to ~7-10% in recent years, thanks largely to fixed-bearing designs [18]. When lateral UKA does fail, conversion to TKA is usually performed; this strategy has good outcomes and a lower re-revision rate than trying another UKA. In summary, careful surgical technique and component positioning (checked via imaging) can minimize failures, and registry data indicate that well-executed lateral UKA has very high long-term survival [18,20].

Historical controversies, limitations, and future directions

Lateral UKA was historically underused due to technical challenges and early prosthesis shortcomings. Early studies reported high revision rates with lateral mobile-bearing UKA, leading many surgeons to favor TKA for lateral disease. However, contemporary evidence has overturned much of that skepticism. Modern implant designs (fixed-bearing) and better surgical planning have greatly improved outcomes [17,18]. Improved understanding of knee kinematics and careful patient selection have clarified indications. For example, obesity and younger age used to be viewed as contraindications, but recent systematic reviews show that patients with high BMI or even under 60 years old can have satisfying outcomes after lateral UKA [16,17]. In fact, Kennedy et al. explicitly noted that body weight and patellofemoral joint status were not contraindications in their lateral UKA series [16]. These developments represent a “renaissance” of lateral UKA, making it a rational choice for appropriately selected patients with isolated lateral OA. While current registry and cohort data are encouraging, further prospective studies and randomized clinical trials are needed to refine indications, optimize alignment targets, and define the long-term role of lateral UKA in contemporary knee arthroplasty. A summary of the major findings for each domain is included in Table 1.

**Table 1:** Key findings in each domain and their implications UKA: Unicompartmental knee arthroplasty; ACL: anterior cruciate ligament; OA: osteoarthritis; TKA: total knee arthroplasty

Domain	Key Findings	Clinical/Research Implications
Epidemiology & Utilization	UKA accounts for 8-10% of knee arthroplasties in the U.S., with a marked increase over the past decade. Lateral UKA remains uncommon, accounting for 5-7% of all UKAs [9].	Reflects growing acceptance of UKA overall, but persistent underutilization of lateral UKA due to technical complexity and historical concerns.
Cost-Effectiveness	Decision-analysis models suggest UKA is cost-effective compared to TKA when implant survivorship approaches that of TKA, with favorable QALY gains [12].	Supports selective use of lateral UKA in appropriately indicated patients from a health-economics perspective.
Implant Design Evolution	Early mobile-bearing lateral UKAs had high failure rates, largely due to bearing dislocation. Modern fixed-bearing designs show significantly improved survivorship (>90–95% at mid- to long-term follow-up) [17,18].	Fixed-bearing implants are now preferred for lateral UKA, reducing revision risk and improving reproducibility [4].
Patient Selection & Indications	Isolated lateral OA with intact ACL and correctable valgus remains essential. Age, BMI, and mild patellofemoral degeneration are no longer absolute contraindications [16,19].	Broadens candidacy for lateral UKA while emphasizing careful clinical and radiographic selection.
Lateral vs Medial UKA	Clinical outcomes and survivorship of lateral UKA are comparable to medial UKA when performed for correct indications [20].	Counters earlier perceptions that lateral UKA is inherently inferior.
UKA vs TKA	UKA offers faster recovery, more natural kinematics, and fewer early complications, but has higher long-term revision rates than TKA [18,22].	Highlights the trade-off between early functional benefits and revision risk.
Failure Modes	The most common cause of late failure is progression of OA in untreated compartments. Aseptic loosening and bearing-related issues are less frequent with modern implants [19,20].	Reinforces the importance of alignment, ligament balance, and patient selection.
Imaging-Based Failure Analysis	Radiographs, CT, fluoroscopy, and MRI provide complementary assessment of alignment, rotation, loosening, kinematics, and OA progression. CT is particularly valuable for rotational alignment analysis [7,38].	Imaging is central to diagnosing failure mechanisms and guiding revision strategy.
Surgical Technology	Robotic-assisted lateral UKA improves component positioning accuracy and shows excellent long-term survivorship, though clear superiority over expert conventional surgery remains unproven [23].	Technology may enhance precision but should complement (not replace) surgical principles.

## Conclusions

Lateral UKA has evolved into a reliable treatment option for appropriately selected patients with isolated lateral compartment disease. While overall utilization of UKA has increased in recent years, lateral UKA has historically remained underused, largely influenced by earlier studies reporting inferior outcomes and higher revision rates. Contemporary evidence shows that modern fixed-bearing implants and improved surgical techniques have led to durable long-term survivorship and favorable outcomes that are comparable to medial UKA, and in carefully selected patients, TKA. Analyzing implant failure with imaging further supports these findings by enabling objective assessment of implant performance, showing the importance of careful patient selection and precise component positioning. Ongoing refinements in surgical techniques and implants continue to expand and refine the indications for this once-controversial procedure.
